# High HIV Incidence among Persons Who Inject Drugs in Pakistan: Greater Risk with Needle Sharing and Injecting Frequently among the Homeless

**DOI:** 10.1371/journal.pone.0081715

**Published:** 2013-12-16

**Authors:** Rab Nawaz Samo, Arshad Altaf, Ajmal Agha, Omrana Pasha, Shafquat Rozi, Ashraf Memon, Saleem Azam, Meridith Blevins, Sten H. Vermund, Sharaf Ali Shah

**Affiliations:** 1 Bridge Consultants Foundation, Karachi, Pakistan; 2 Department of Community Health Sciences, Aga Khan University, Karachi, Pakistan; 3 Sindh AIDS Control Programme, Karachi, Pakistan; 4 Vanderbilt Institute for Global Health and Departments of Biostatistics & Pediatrics, Vanderbilt University School of Medicine, Nashville, Tennessee, United States of America; 5 Pakistan Society, Karachi, Pakistan; Alberta Provincial Laboratory for Public Health/University of Alberta, Canada

## Abstract

**Background:**

The incidence of HIV among persons who inject drugs (PWIDU) has fallen in many nations, likely due to successes of clean needle/syringe exchange and substance abuse treatment and service programs. However in Pakistan, prevalence rates for PWID have risen dramatically. In several cities, prevalence exceeded 20% by 2009 compared to a 2003 baseline of just 0.5%. However, no cohort study of PWID has ever been conducted.

**Methods:**

We enrolled a cohort of 636 HIV seronegative PWID registered with three drop-in centers that focus on risk reduction and basic social services in Karachi. Recruitment began in 2009 (March to June) and PWID were followed for two years. We measured incidence rates and risk factors associated with HIV seroconversion.

**Results:**

Incidence of HIV was 12.4 per 100 person-years (95% exact Poisson confidence interval [CI]: 10.3–14.9). We followed 474 of 636 HIV seronegative persons (74.5%) for two years, an annual loss to follow-up of <13 per 100 person years. In multivariable Cox regression analysis, HIV seroconversion was associated with non-Muslim religion (Adjusted risk ratio [ARR] = 1.7, 95%CI:1.4, 2.7, p = 0.03), sharing of syringes (ARR = 2.3, 95%CI:1.5, 3.3, p<0.0001), being homeless (ARR = 1.7, 95%CI:1.1, 2.5, p = 0.009), and daily injection of drugs (ARR = 1.1, 95%CI:1.0, 1.3, p = 0.04).

**Conclusions:**

Even though all members of the cohort of PWID were attending risk reduction programs, the HIV incidence rate was very high in Karachi from 2009–2011. The project budget was low, yet we were able to retain three-quarters of the population over two years. Absence of opiate substitution therapy and incomplete needle/syringe exchange coverage undermines success in HIV risk reduction.

## Introduction

The HIV epidemic in Pakistan began in 1986 when a foreign sailor died of AIDS in Karachi; local transmission was documented in 1987 [1], [2]. For almost 20 years, most cases were imported [3]. In 2003, the prevalence of HIV among persons who inject drugs (PWID; also known as injection drug users) was 0.6% in Karachi, Pakistan's major port and largest city [4]. The first outbreak of HIV among PWID was documented in June 2004 in Larkana, a city of half a million persons in Sindh Province, when 17 of 183 (9.3%) PWID tested HIV seropositive [5], [6]. From 2003 to 2007, HIV prevalence among PWID in Karachi rose from 0.3% to 23% in 2004 and has reached 42% in 2011 [6], [7]. The findings of 2011 national surveillance showed an extremely high prevalence in other cities of Pakistan [7]. In Faisalabad and DG Khan in Punjab province the prevalence has reached 52.5% and 49.6% respectively. In Sargodha the second documented HIV outbreak was reported in 2007 when out of 400 recruited PWID more than half (51.3%) were confirmed positive [8]. In Gujrat, a relatively small town of Punjab province, 46.2% PWID were positive [7]. This is the same town where in 2008 in an HIV screening camp, out of 246 persons fully 35.8% were HIV positive. This new outbreak attracted attention and the Field Epidemiology and Laboratory Training Program of CDC conducted an investigation. Injection of illicit drugs was the principal cause [9].

PWID are now the principal drivers of the HIV epidemic in Pakistan, yet the quality of harm reduction services remains far below acceptable standards. The first harm reduction program was initiated in Karachi in 2000 and with passage of time at least three non-governmental organizations (NGOs) had been providing services in this megacity of over 20 million persons. However, NGO program coverage has remained low and has not reached more than 16% of the target estimated population of PWID [10]. There are no opiate/opioid substitute therapy (OST) programs in Karachi for treatment of addiction. The available choices are limited to 2–4 weeks of detoxification via “cold turkey” methods, ameliorated only with analgesics. There is dearth of data on actual relapse rates but the program managers of harm reduction program quote a relapse rate figure of >80% among PWID (at least one has told us that it is 100%).

Risk factors and prevalence of HIV and other blood-borne infections among PWID are documented in diverse Pakistani venues [4]–[37]. However, no incidence study has been published and it is not known to what extent PWID could be followed successfully in Pakistan. We measured HIV seroincidence and factors associated with HIV seroconversion among PWID enrolled in three drop-in centers in Karachi for two years.

## Methods

### Setting

The study was conducted in Karachi, the nation's major seaport and economic hub, most populous city, and the capital of Sindh Province. The population is estimated at more than 20 million persons with high diversity of ethnic groups [38]. Karachi has an estimated 16,500 PWID [17], though this may be a minimum estimate given sampling uncertainties. We conducted our study at three drop-in centers that provide basic harm reduction and social services to PWID exclusive of opiate substitution therapy; the NGO that runs the drop-in centers has been working with PWID since 1994.

### Sample size

The sample size was determine keeping in mind that the persons using the needle exchange services with fidelity might have an HIV seroincidence of 5% per year compared to a higher rate of 12% per year in the lower-user group. Using Open-Epi, both the Fleiss and the Kelsey approaches required 250 persons per group (higher and lower program utilizers). Since loss to follow up is very high among IDUs in such programs we assumed that 30% of IDUs would be lost to follow up over two years, suggesting that we would need 650 persons. Since we had lower-than-expected loss to follow-up, our 636 study participants recruited into our study who were enrolled in the program during March–June 2009 were adequate to address the research question with a two-sided significance level of 95% and a statistical power of 80%.

### Design

We recruited 636 injection drug users and followed them until they were lost-to-follow up or the end of the two year study from March 2009 to June 2011, for the estimation of incidence rate and risk factors associated with HIV seroconversion. We excluded eight persons at baseline who were unable to understand the study objectives due to disabilities that precluded provision of informed consent.

### Follow up visits and tracking the study participants

The follow up visits by the outreach workers were on daily basis. Three investigators (RS, AA and SAS) visited on a monthly basis. The participants were tracked by the staff of the risk reduction programs. The “in-charges” of the drop-in centers were in touch with the participants during whole study period, though some were lost to follow-up.

### Techniques used to track the participants

The participants were tracked by the staff of the risk reduction programs. The “in-charges” of the drop-in centers were in touch with the participants during whole study period, though some were lost to follow-up.

### Laboratory testing

The initial rapid test screening of the study participants was done with the SD BIOLINE HIV-1/2 3.0 test™ (Standard Diagnostics, Inc., Korea). The clients reactive on the rapid test gave 5 mL venous blood for confirmatory testing at the Referral Laboratory of the Sindh AIDS Control Program, Civil Hospital Karachi, for confirmation using the UNAIDS/WHO/Government of Pakistan HIV Testing strategy of positivity on two different ELISAs (Murex HIV Ag/Ab Combination™, Abbott-Murex. Ltd, and AniLabsystems HIV EIA™ kit, LabsystemsMultiscan MS, Labsystems, Finland). Dual ELISA confirmed PWID were assessed for combination antiretroviral therapy eligibility by clinical assessment and CD4+ cell counts in the Reference Lab. Only persons seroreactive on the rapid test and two confirmatory tests were considered to be HIV seroconverters. All confirmed HIV seropositive clients were enrolled into the Antiretroviral Treatment Center of the Sindh AIDS Control Program for care and antiretroviral treatment if WHO and national guideline eligibility for therapy was met (CD4+ cells <350/µL and/or WHO clinical status 3 or 4).

### Ethical considerations

Ethics Review Board of Aga Khan University specifically approved this study. Privacy and confidentiality of the participants were assured at all levels of study. Data were password protected to which only the principal investigator had access. Written informed consent was obtained from all eligible study participants. The study participants had the right to refuse blood tests or interviews and could withdraw from the study at any time without compromising their access to the services of the drop-in centers. No monetary incentive was offered. The project budget from the Fogarty International Center was US$5000 for two years, along with a research training stipend for the principal investigator (RNS). With the knowledge and consent of the client, results for HIV positive PWID were shared with the NGO director such that HIV care services could be offered.

### Data collection

The data were collected through a field-tested questionnaire, developed in English and translated into Urdu. The data collectors were trained interviewers who had previous experience of working with the PWID. The data were collected on socio-demographics, risk behaviors, drug history, and knowledge of HIV/AIDS.

### Statistical analyses

#### Data entry and analysis

Data were entered into EpiInfo™version 6.0 (Centers for Disease Control and Prevention) and analyzed using the Statistical Package for Social Sciences (SPSS™) version 17 and OpenEpi™ version 2.3.1. Exact confidence intervals were calculated using R statistical software, version 2.13.1 (available at: http://www.R-project.org; R Foundation for Statistical Computing, Vienna, Austria). Data were double entered by the two independent data entry staff and discrepant entries were checked for accuracy and resolution.

Frequencies were calculated for categorical data; means and standard deviations were calculated for the continuous variables. The estimated date of seroconversion was determined as the mid-point between last negative and first positive test. The incidence rate was defined as the total number of incident cases per 100 person-years contributed. Both confidence intervals and sensitivity analyses were assessed with exact Poisson methods. Sensitivity analyses were based on plausible “high-low” assumptions for incidence among lost to follow-up subjects. All biologically or behaviorally important variables were selected for univariate analyses to estimate unadjusted risk ratios (RR) and 95% confidence intervals (95%CI). All variables associated with seroconversion at p≤0.2 were included in a multivariable Cox regression model. Confounding was assessed by a 10% change in beta coefficient of any independent variable in the model.

## Results

All participants were seronegative at the beginning of the study, as per the study design. The retention rate of the PWID in the program was 474 of 636 (74.5%) over the 2 year period, a loss-to-follow-up rate of just less than 13 per 100 person years. Excluding the 162 men lost to follow-up 2 years, study we had confirmed 118 (24.9%) HIV seropositive and 356 (75.1%) HIV Seronegative PWID (**Table 1**), at the end of the study (24 month follow-up).

**Table 1:** Descriptive characteristics of 474 injection drug users followed for two years in a Karachi, Pakistan cohort.

Characteristic	n (%)
**Age (mean in years ± S.D.)**	30.0±9.0
**HIV serostatus at the end of the cohort period**	
Positive	118 (24.9)
Negative	356 (75.1)
**Source of registration in the study**	
Outreach	361 (76.2)
Others^a^	113 (23.8)
**Religion**	
Muslim	418 (88.2)
Christian	54 (11.4)
Hindu	2 (0.4)
**Ethnicity**	
Sindhi	225 (47.5)
Non Sindhi^b^	249 (52.5)
**Education**	
Some formal education	130 (27.4)
No formal education	344 (72.6)
**Professional skills**	
None	280 (59.1)
Some	194 (40.9)
**Currently sharing of needles/syringes**	
Yes	101 (21.3)
No	373 (78.7)
**History of arrest, ever**	
No	222 (46.8)
Yes	252 (53.2)
**Physical disability**	
Yes	14 (3.0)
No	460 (97.0)
**Marital status**	
Married	220 (46.4)
Un-married	254 (53.6)
**Monthly income in rupees** c	
<5000	277 (58.4)
>5000	197 (41.6)
**Last syringe used**	
New	422 (89.0)
Used	52 (11.0)
**Source of syringe**	
Drop-in-centers (DIC) and mobile service units (MSU)	160 (33.8)
Others^d^	314 (66.2)
**Know about HIV/AIDS**	
Yes	24 (5.1)
No	450 (94.9)
**Know about dangers of needle sharing**	
Yes	21 (4.4)
No	453 (95.6)
**Ever treated for sexually transmitted infection**	
Yes	9 (1.9)
No	465 (98.1)
**Knows that HIV spreads through unprotected sex**	
Yes	49 (10.3)
No	425 (89.7)
**Typical location of drug use**	
Home	69 (14.6)
Street	405 (85.4)
**Living in streets**	
No	203 (42.8)
Yes	271 (57.2)
Frequency of daily drug use (mean and S.D^e^)	3.7±1.4

^a^ Government organization, non-governmental organization, community, and friends.

^b^ Non-Muslim included 54 Christians and 2 Hindus vs. 418 Muslims.

≈US$ 56.^c^ Approximately 90 rupees per US dollar in this time period, such that 5000 rupees

^d^ Pharmacy, friends, and hospital garbage.

^e^ Drop-in center (DIC) and mobile service unit (MSU).

HIV incidence was 12.4 per 100 person-years among PWID enrolled, with 95% exact Poisson CI: 10.3–14.9 (Table 2). Over a variety of plausible assumptions for the men lost to follow-up (LTFU), sensitivity analyses suggested our study to be robust in its estimate of incidence. If the two extremes are used, one might assume 17.4% of LTFU were HIV-infected (30% lower than observed), such that the HIV incidence would be 11.5 per 100 person-years (95% CI: 9.69–13.5). In contrast, one might assume 32.4% of LTFU were HIV-infected (30% higher than observed), such that the HIV incidence would be 13.4 per 100 person-years (95% CI: 11.4–15.5).

**Table 2:** Sensitivity analysis of the estimates for HIV incidence per 100 person-years (py) with 95% exact Poisson confidence intervals (CI), considering persons lost to follow-up (LTFU) in a variety of plausible “high-low” scenarios for their unknown incidence.

Scenario	Events	Person-time	Incidence per 100 py	Standard error	Exact lower 95% CI	Exact upper 95% CI
**Do not adjust for LTFU (use observed data only)**	118	948	12.4	1.15	10.3	14.9
**Assume 17.4% of LTFU are HIV+ (30% lower than observed)**	146	1272	11.5	0.95	9.69	13.5
**Assume 20% of LTFU are HIV+ (same as observed)**	150	1272	11.8	0.96	9.98	13.8
**Assume 24.9% of LTFU are HIV+ (same as observed)**	158	1272	12.4	0.99	10.6	14.5
**Assume 30% of LTFU are HIV+**	167	1272	13.1	1.02	11.1	15.3
**Assume 32.4% of LTFU are HIV+ (30% higher than observed)**	170	1272	13.4	1.03	11.4	15.5
**HIGH-LOW RANGE OVER ALL ASSUMPTIONS**			11.5–13.4		9.7–15.5	

The mean age of PWID in one of the three drop-in risk reduction programs was 30.0±9.0 years. Active outreach was the major source of enrollment (n = 361, 76.2%) into the study. Most clients (n = 418, 88.2%) were Muslims, while Christians represented 11.4% (n = 54) and Hindus 0.4% (n = 2). No formal education was reported by 344 (72.6%) of participants and 280 (59.1%) reported having no work skills. About one of five participants reported sharing needles/syringes (n = 101, 21.3%). Most participants (n = 252, 53.2%) had a history of prior arrest. Most participants were destitute with 277 (58.4%) having an income <5000 rupees per month (equivalent in 2009 to US$75/month). About half of the participants (n = 254, 53.6%) were unmarried. Most participants (n = 282, 59.5%) reported 2–4 episodes of drug injecting/day. Most participants (n = 314, 66.2%) were getting syringes from sources other than the drop-in centers or the mobile service units.

### Predictors of seroconversion

Eight factors were associated with seroconversion among the 474 persons in univariate analyses (Table 3) and were included in the multivariable Cox regression analysis: sharing of syringes (p<0.0001), homelessness(p = 0.008), non-Muslim religion (p = 0.02), source of syringes other than the drop-in centers or mobile service units (p = 0.02), daily frequency of injecting drugs (p = 0.03), monthly income <PKR 5,000 (p = 0.04), physical disability (p = 0.06), and source of registration other than outreach (p = 0.2). In multivariable analysis, four factors remained as significant predictors of seroconversion (Table 4): sharing of syringes (ARR = 2.3, 95%CI: 1.5, 3.3, p<0.0001); non-Muslim religion (ARR = 1.7, 95%CI:1.4, 2.7, p = 0.03); being homeless (ARR = 1.7, 95%CI:1.1, 2.5, p = 0.009); daily frequency of injecting drugs (ARR = 1.1, 95%CI:1.0, 1.3, p = 0.04).

**Table 3:** Univariate regression analysis of factors associated with seroconversion in a cohort of 474 injection drug users followed for two years in Karachi, Pakistan.

Variable	RR	95% CI	p-value
**Source of registration**			
Others^a^ (vs Outreach)	1.3	0.9, 1.9	0.2
**Religion**			
Non Muslims^b^ (vs. Muslim)	1.7	1.1, 2.7	0.02
**Ethnicity**			
Non Sindhi^c^ (vs. Sindhi)	0.9	0.6, 1.3	0.6
**Education**			
Illiterate (vs. Literate)	1.2	0.8, 1.9	0.4
**Currently Sharing of needles/syringes**			
Yes (vs. No)	2.4	1.7, 3.5	<0.0001
**History of arrest**			
Yes (vs. No)	1.0	0.7, 1.5	0.9
**Physical disability**			
Yes (vs. No0	2.0	0.9, 4.4	0.06
**Marital status**			
Unmarried (vs. Married)	1.0	0.7, 1.4	0.9
**Monthly income Pakistani rupees** d			
<5000 (vs. ≥5000)	1.5	1.0, 2.1	0.04
**Source of syringes/needles**			
Other^e^ (vs. DIC & MSU)^f^	1.6	1.1, 2.5	0.02
**Ever treated for sexually transmitted infection**			
Yes (vs. No)	1.3	0.4, 4.2	0.6
**Home**			
Homeless (vs. having home)	1.7	1.2, 2.5	0.008
**Daily frequency of injections (#/day)**	1.1	1.0, 1.3	0.03

^a^ Government organization, non-governmental organization, community, and friends.

^b^ Non-Muslim included 54 Christians and 2 Hindus vs. 418 Muslims.

^c^ Non-Sindhis included Pathan, Punjabi, Hindko, Kashmiri, and Bengali.

≈US$ 56.^d^ Approximately 90 rupees per US dollar in this time period, such that 5000 rupees

^e^ Pharmacy, friends, and hospital garbage.

^f^ Drop-in center and mobile service unit.

**Table 4:** Multivariable Cox regression analysis for associations with HIV seroconversion in a two-year cohort of injection drug users in Karachi, Pakistan (n = 474).

Variable	Adjusted Risk Ratio	95% CI	p-value
**Source of registration**			
Others^a^ (vs Outreach)	1.1	0.8, 1.8	0.4
**Religion**			
Non Muslims^b^ (vs. Muslim)	1.7	1.4, 2.7	0.03
**Currently Sharing of needles/syringes**			
Yes (vs. No)	2.3	1.5, 3.3	<0.0001
**Physical disability**			
Yes (vs. No0	1.7	0.8, 3.7	0.2
**Monthly income Pakistani rupees** c			
<5000 (vs. ≥5000)	1.2	0.9, 2.3	0.06^f^
**Source of syringes/needles**			
Other^d^ (vs. DIC & MSU)^e^	1.5	0.9, 2.3	0.009
**Daily frequency of injections (#/day)**	1.1	1.0, 1.3	0.04

^a^ Government organization, non-governmental organization, community, and friends.

^b^ Non-Muslim included 54 Christians and 2 Hindus vs. 418 Muslims.

≈US$ 56.^c^ Approximately 90 rupees per US dollar in this time period, such that 5000 rupees

^d^ Pharmacy, friends, and hospital garbage.

^e^ Drop-in center (DIC) and mobile service unit (MSU).

= 0.046).^f^ If source of registration, physical disability, and monthly income, are excluded from the above Cox regression to achieve a more parsimonious model, then the effect sizes are similar and source of syringes becomes a significant predictor (p

## Discussion

The high incidence rate documented in our cohort (12.4 per 100 person-years) is likely a minimum HIV incidence rate for PWID in Karachi, given that, for ethical reasons, the PWID recruited were followed within the context of local risk reduction programs. The incidence rate among active PWID not attending the drop-in centers is likely even higher. PWID in Sichuan Province (China), St. Petersburg (Russia), Xinjiang Province (China), and Bangkok (Thailand) have been followed with seroincidence rates of 3.2, 4.5, 8.8, and 5.8 per 100 person-years respectively [36]–[39]. Hence our incidence data may be among the highest yet reported in Asia or Eastern Europe where PWID continue to play a dominant role in driving HIV transmission.

Our study was conducted at exceedingly low cost; we think the 13% annual loss to follow-up could be reduced if the cohort were being followed in a funded clinical trial with research resources. Nevertheless, our retention rate was better than we anticipated, suggesting that drop-in centers might be an excellent venue for engagement of high risk PWID in order to offer them locally available harm reduction. Mitigation of IDU medical and social consequences would be an expected favorable consequence of expanding risk reduction service coverage to all PWID, if high quality and coverage could be maintained.

In Karachi's last census (1998), Christians represented 2.4% of the population, but current estimates for a Karachi in 2011 are that Christians represent <1% of Karachi's population. Yet Christians were disproportionately represented among PWID, 11.4% in our cohort, and also were overrepresented in seroconverters (Table 1). This finding was noted alongside more familiar risks for HIV acquisition: needle/syringe sharing, homelessness, and higher frequency of drug injection. PWID who belong to a religious and cultural minority might face real or perceived challenges in access and utilization of the program services, though all our subjects were in the same risk reduction program. They might be discriminated against in society, e.g., in job seeking and have other difficulties in social interaction. They might inject drugs in a closed group having similar socio-cultural characteristics [40]. The risk of HIV might increase in such a group when someone is infected with HIV, spreading the infection in the whole group due to sharing of needles and syringes.

Studies from around the world have shown the importance of needle/syringe sharing for HIV acquisition [41], [42]. Living on the streets exposes PWID to health hazards, sexual abuse, and more risky behaviors as compared to those who live in homes. Many Karachi homeless PWID live near garbage dumps where food and needles can be scrounged. The experience of our drop-in care providers suggests that PWID living in streets are prone to sell sex for drugs, free injections, or money. We found, too, that as the frequency of injecting drugs per day increases, the risk of HIV acquisition increases, a logical relationship also seen in Vancouver, Canada [41].

The high incidence rate among clients of harm reduction program should be alarming for Pakistani and global public health and welfare officials and other stakeholders. In Pakistan, conservative socio-cultural norms often fail acknowledge IDU as a priority concern; this limits prevention investments such that program coverage and services of risk reduction and drug treatment programs in Karachi are low. Opiate agonist therapy is almost completely unavailable, for example, and the only buprenorphine pill available is such a low dose as to be almost homeopathic. Underfunding compromises the quality and quantity of outreach services and the full implementation of risk reduction programs. Quality improvement feedback mechanisms based on modern monitoring and evaluation programs are largely missing from risk reduction programs [17], [18]. All these program deficits contribute to the exceedingly high incidence rates.

Barriers to scale-up of needle/syringe exchange programs and of opiate/opioid substitution therapy are many [43]–[45]. Lack of political will, lack of funds (against other perceived needs), stigma biasing policy makers against assistance for risk reduction about PWID, and legal obstacles are a few of these. Unless scaling up of risk reduction programs for PWID, Pakistan cannot expect any of the successes seen in so much of Asia in reduced incidence among PWID [46], [47]. HIV testing must be expanded in this population, along with facilitated linkage to HIV care and social services, both to benefit infected PWID and to reduce their infectiousness to others [48]–[51]. PWID are a risk to the general population given that they mix with female and male sex workers, prisoners, and spouses, as well as contribute blood for pay to illicit blood collection agencies [52], [53], [62]. New highways better connect cities within Sindh Province as well as to other regions in Pakistan where HIV prevalence has been lower, risking further spread of virus [54]–[56].

The principal strength of our study is that it is unique, the first-ever documentation from Pakistan of the incidence rate of HIV among PWID. True seroincidence rates are hard to come by, given the complexity of cohort assembly and follow-up. Given that our research budget was low, we believe that a 74% follow up rate over two years was encouraging. A limitation is that our study was a prospective study within a risk reduction program context, so we cannot generalize our findings to persons not attending the drop-in centers and those not in Karachi. We do not know the incidence rates for those who were lost to follow up and were not retained in the harm reduction program, but it is reasonable to assume that they might have been even higher than among men in the risk reduction programs. Another limitation was that we did not back-translate our questionnaires. However, the translators were bilingually fluent and the investigators, also bilingual, found the translations to have full fidelity with the original in English, so we do not believe that this affected our results. We mention this limitation in the Discussion section.

The HIV incidence rate among PWID enrolled in harm reduction programs at Karachi is alarming, perhaps among the highest in the world in the 2009–2011 time period. Timely and appropriate programmatic initiatives are needed to avert both increased disease burden and expanded transmission to the general population [57]–[61]. More work is needed in risk of hepatitis transmission and in the molecular epidemiology of IDU-related transmission [62]–[64]. Coverage of and fidelity (adherence) to clean needle use in the context of needle and syringe exchange are critical elements needing quality improvement [65]–[73]. Research into innovative, promising improvements in risk reduction, drug treatment, and HIV prevention are feasible in this context, given our success in cohort implementation and the high documented seroincidence rates. Expanded needle/syringe exchange, detoxification and rehabilitation services, and use of opioid agonist therapy in harm reduction and drug treatment programs can play an instrumental role in mitigating the dual burden of HIV infection and drug addiction in Pakistan [45], [74]–[76].
